# Transcriptomic and metabolomic analyses unravel the different pathogenic mechanisms of *Ustilaginoidea virens* in indica and japonica rice

**DOI:** 10.3389/fmicb.2025.1680221

**Published:** 2025-10-17

**Authors:** Rongtao Fu, Yu Chen, Cheng Chen, Jian Wang, Liyu Zhao, Daihua Lu

**Affiliations:** ^1^Institute of Plant Protection, Sichuan Academy of Agricultural Science, Chengdu, China; ^2^Key Laboratory of Integrated Pest Management on Crops in Southwest, Ministry of Agriculture, Chengdu, China

**Keywords:** *Ustilaginoidea virens*, indica rice, japonica rice, transcriptome, metabolome

## Abstract

Rice false smut (RFS) caused by the fungal pathogen *Ustilaginoidea virens* (Cook) produces high yield losses in rice. Rice varieties differ in their resistance to RFS. However, the pathogenic mechanism of the fungus *U. virens* in different varieties remains unclear. In our study, transcriptome and metabolome analyses were performed on *U. virens* after 5 and 7 days after infection in indica and japonica rice to reveal different pathogenic mechanisms. Interestingly, we discovered that the average number of diseased grains of the susceptible variety “GuiChao2” (indica rice) was higher than that of the susceptible variety “Zhejing99” (japonica rice) under the same inoculation conditions. In all, 6,073, 5,795, 4,251, and 5,978 differentially expressed genes (DEGs) were shared among the four infected rice compared with the control group. In this study, there were differences in the types and quantities of transcription factors in *U. virens* after infection of indica and japonica rice. A Kyoto Encyclopedia of Genes and Genomes analysis showed that the DEGs involved in the mitogen-activated protein kinase signaling pathway, autophagy–yeast, lysine biosynthesis, phenylalanine metabolism, and tryptophan metabolism responded to infection, and the expression patterns of key regulatory genes, including *STE*, *ATG*, *CYP*, and *LYS* differed after infection indica and japonica rice. The results of a metabolome analysis indicated that the differentially accumulated metabolites (DAMs) mainly were significantly enrichment in pathways included amino acids, lipids, and nucleosides, and the accumulation patterns between infected indica and japonica rice differed. Furthermore, the combined transcriptome and metabolome analysis revealed that different DAMs regulated by different DEGs produced variations in the pathogenicity of *U. virens* infection in indica and japonica rice. Hence, this study provides insight into the molecular mechanisms related to *U. virens* infection in different rice varieties.

## 1 Introduction

The fungus *Ustilaginoidea virens* is the causal agent of rice false smut (RFS), which mainly infects rice panicles at the rice booting stage (Tanaka et al., 2008). As the mycelium continues to invade the plant, the reproductive parts of a flower, including the anthers, filaments, stigma and placenta, become completely enveloped by the mycelium, gradually forming a rice smut ball (Ashizawa et al., 2013). This reduces the rice yield and produces large quantities of mycotoxins that are harmful to humans and animals (Sun et al., 2020). In recent years, with the large-scale production of high-yield rice and hybrid rice, excessive use of chemical fertilizers, and global climate changes, RFS has gradually become a major disease in rice-growing areas worldwide. In China, the degree to which RFS occurs varies greatly from year to year, with a distinct intermittent outbreaks and epidemics (Rao et al., 2018).

It was reported that most rice varieties used in production lack vertical resistance to RFS. Different varieties have different levels of resistance to RFS disease. After conducting resistance identification on different types of varieties, it was found that the resistance level of indica rice germplasm to RFS was higher than that of japonica rice (Chen et al., 2019; Fu et al., 2022). This may be because the genetic background of indica rice contains more genes related to resistance to RFS than that of japonica rice, with these genes exhibiting relatively stable performance under different environmental conditions (Duan et al., 2024). It is also possible that the differences in susceptibility to RFS are caused by the different panicle traits between indica rice and japonica rice (Song et al., 2023). However, it is still unknown whether the pathogenicity of *U. virens* itself differs between indica rice and japonica rice, and whether this causes the resistance of indica rice and japonica rice to RFS to differ.

With the continuous development of omics technologies, multi-omics analysis is now widely applied to interpret the biological mechanisms of plants, and microorganisms, among which transcriptomics and metabolomics are relatively mature, in-depth, and simple technologies (Sana et al., 2010; Lu et al., 2022). The combined analysis of transcriptomics and metabolomics is an experimental means of obtaining comprehensive profile of genes and metabolites. Transcriptomics can reveal differentially expressed genes (DEGs) under various conditions, while the analysis of metabolites, as the end products of gene transcription and translation, serves as a bridge between genes and phenotypes. The comprehensive analysis of these two omics can uncover the causes and results of internal changes in organisms, revealing both the lock and key pathways of changes in genes and metabolites and important regulatory networks. The combined analysis of transcriptome and metabolome has been applied to study the stress and growth of pathogenic fungi, such as *Rhizopus oryzae,* and *Candida albicans* (Liboro et al., 2021; Xu et al., 2018). In our previous work, we also used multi-omics analysis to study the pathogenic mechanism of *U. virens* on rice (Fu et al., 2023).

However, the molecular and metabolic pathways that *U. virens* affects in indica and japonica rice are still unclear. In the present study, we performed a multi-omics analysis using the transcriptome and metabolome of infected indica and japonica rice strains to reveal the different pathogenic mechanism of *U. virens* in this strain. These results showed that the DEGs involved in the mitogen-activated protein kinase signaling pathway, autophagy–yeast, lysine biosynthesis, phenylalanine metabolism, and tryptophan metabolism responding to infection. Moreover, the expression patterns of key regulatory genes, including *STE*, *ATG*, *CYP*, and *LYS*, differed between infected infection indica and japonica rice.

## 2 Materials and methods

### 2.1 Fungal inoculation and plant materials

The fungus *U. virens* PXD25 used in this experiment was isolated and preserved by the Plant Protection Institute of Sichuan Academy of Agricultural Sciences. The strain was cultured on potato sucrose agar medium, and mycelium disks were placed in potato sucrose broth (PSB). The cultures were incubated at 26 °C and 130 rpm for 10 days. The conidia and mycelia were collected for inoculation.

The susceptible indica rice “Guichao2” and susceptible japonica cultivar “Zhejing99” were used in this study. The rice plants were grown in a controlled greenhouse at the experimental base of the Sichuan Academy of Agricultural Sciences. The entire growth period was managed according to conventional cultivation management. The inoculation method described by Fu et al. (2024) was used with minor modifications. At the seventh stage of panicle development, approximately 5 mL of a conidial suspension at a concentration of 1 × 10^6^ mL^−1^ was injected into rice panicles. The controls were injected with PSB in all experiments. After inoculation, the rice plants were maintained at 25 °C /30 °C (night/day), covered with a sunshade net, and automatically sprayed with water for 5 min every 2 h to maintain an environment at a relative humidity >85% for 4 d. Samples of pathogens before (CK) and after infection with mycelium were collected, immediately frozen in liquid nitrogen, and stored at −80 °C for later use. We chose two time points for sampling at which mycelial symptoms appeared, (5 and 7 d). The two samples collected from the indica rice infection site at the two time points were designated X1 and X2, and the two samples collected from the japonica rice infection site were designated J1 and J2.

### 2.2 Transcriptome sequencing and data analysis

The total RNA of each sample was extracted using TRIzol (Aidlab Biotechnologies, Beijing, China) following the manufacturer’s protocol. There were three biological replicates per sample group. The integrity and quantity of the isolated RNA were determined using 1.5% agarose gel and an Agilent 2100 bioanalyzer (Agilent Technologies, Santa Clara, CA, United States), respectively. The obtained high-quality RNA underwent library construction and sequencing at Novogene Biotechnology Co., Ltd., Beijing, China on a HiSeq™ 2500 platform (150 bp paired end reads).

To analyze the sequencing data, raw reads were filtered to remove low-quality sequences using default parameters. The clean reads were mapped to the reference genome of *U. virens* using HISAT2 (Kim et al., 2015). The gene expression level was calculated using HTSeq software (v 0.5.4) and normalized using the fragments per kilobase per million fragments (Anders and Huber, 2010). DEseq2 was used to analyze differentially expressed genes (DEGs) between the two groups. DEGs were identified by comparing gene expression levels of each strain before and after infection between the CK and X and J groups with the criteria *p* < 0.05 and |log2 fold change| ≥ 1 (Love et al., 2014). The identified DEGs then underwent functional annotation using Gene Ontology (GO) and Kyoto Encyclopedia of Genes and Genomes (KEGG) databases. A *q*-value ≤ 0.05 was considered the threshold for significant enrichment of GO and KEGG pathways based on the DEGs.

### 2.3 Quantitative real-time PCR validation

To validate the DEG results, quantitative real-time PCR (qRT-PCR) was performed on 10 DEGs, with *actin* serving as the internal reference. The primer sets used for qRT-PCR were designed according to the individual gene sequences (Supplementary Table S5). The same RNA sample was used for both RNA-Seq and qRT-PCR. Following the manufacturer’s protocols, the first-strand cDNA was synthesized using a first-strand cDNA synthesis kit (TransScript, Beijing, China). qRT-PCR was performed using Takara SYBR Green (Takara, Dalian, China) in One-Step Real-Time system. The relative gene expression levels were calculated using the 2^−∆∆Ct^ method with triplicate independent samples (Livak and Schmittgen, 2001).

### 2.4 Untargeted metabolomic detection and analysis

To identify differences in metabolites before and after infection of indica and japonica rice by *U. virens*, we chose 15 samples (three in each group, five groups) for metabolomics. Ultra-high performance liquid chromatograph Q Exactive mass spectrometry (UHPLC-QE-MS) was used for untargeted metabolomic profiling. The sample preparation and measurements were performed using the previously reported methods with minor modifications (Ding et al., 2023). Briefly, the samples were ground to powder under liquid nitrogen. Then, 50 mg of the powdered sample was added to a 2 mL EP tube and mixed with 1.0 mL of a methanol/acetonitrile/water solution (2:2:1, v/v). The sample was vortexed, ultrasonicated at a low temperature for 40 min, and left to stand at −20 °C for 15 min. Then, the samples were centrifuged at 4 °C and 15,000 × *g* for 20 min. Finally, the supernatant was filtered through a microporous organic filter membrane (0.22 μm) for analysis using UHPLC. The sample was separated using a Vanquish LC UHPLC and then analyzed by Novogene Co., Ltd. (Beijing, China) using mass spectrometry on a Q Exactive series mass spectrometer (Thermo Fisher Waltham, MA, United States). Electrospray ionization was performed in positive and negative ion modes.

After the mass spectrum data of the metabolites in different samples were obtained by triple quadrupole mass spectrometry, the mass spectrum peaks of all substances were integrated, and the mass spectrum peaks of the same metabolites in different samples were calibrated. The metabolite data were analyzed qualitatively and quantitatively relatively using the mzCloud (https://www.mzcloud.org/), mzVault, and MassList databases. To obtain stable and accurate metabolomic data, Pearson correlation coefficients between quality control (QC) samples were calculated based on the relative quantitative values of metabolites (Rao et al., 2016). Principle component analysis (PCA) was performed using the statistical function prcomp in R software (http://www.r-project.org/accessed on December 16, 2024). Metabolites with a variable in projection (VIP) > 1.0 and *p*-value< 0.05 were considered to be differentially accumulated metabolites (DAMs) for group discrimination (Chen et al., 2022). The KEGG databases were used to annotate the functions of the DAMs, and the metabolic pathways involving the metabolites were identified.

### 2.5 Integrated metabolome and transcriptome analysis

Based on the data from two major omics analyses, the common KEGG pathways of DEGs and DAMs were jointly analyzed. The R cor program in R package was used to calculate the Pearson correlation coefficients of genes and metabolites. The DEGs and DAMs with a Pearson correlation coefficient >0.8 and *p*-value < 0.05 were selected for network maps to reveal molecular interactions between metabolites and genes during the pathogenic process of *U. viren*.

### 2.6 Statistical analysis

All data are expressed as the mean ± SD of three replicates for each treatment. One way analysis of variance (ANOVA) in SPSS 20.0 (IBM, Armonk, NY, United States) was also used for statistical analysis. A *p* < 0.05 indicated a statistically significant difference.

## 3 Results

### 3.1 Symptoms after infection of indica and japonica rice with *Ustilaginoidea virens*

**Figure 1:**
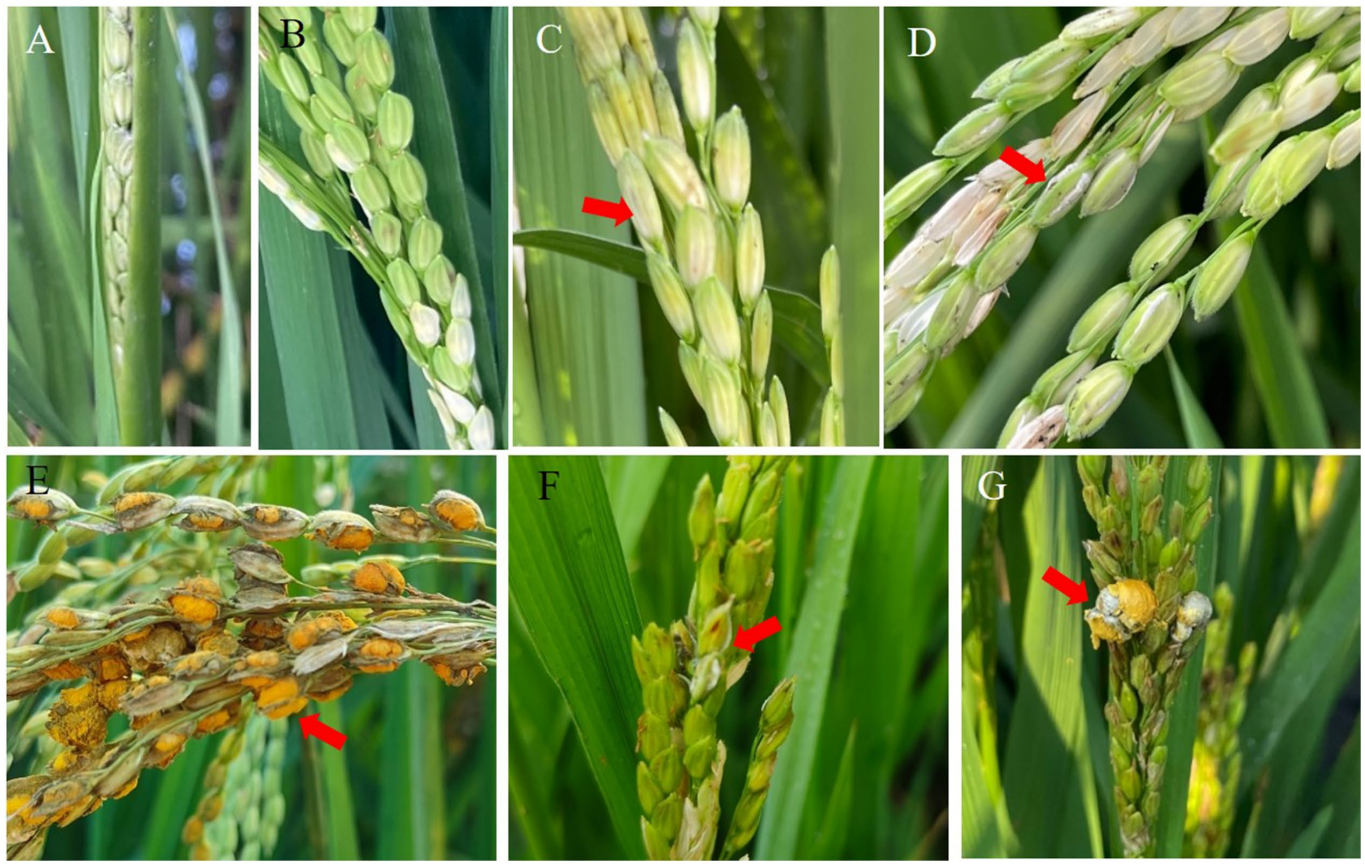
Symptoms of *Ustilaginoidea virens* infection on Guichao2 (indica rice) **(A–E)** and Zhejing99 (japonica rice) **(F–G)** after inoculation.

The panicles of the susceptible indica rice Guichao2 and susceptible japonica cultivar Zhejing99 were inoculated with the pathogen isolate PXD25 at the rice booting stage. The spikelets showed no visible change after 3 days (Figure 1A). On day 5, there were several visible mycelia on the samples (Figure 1B). On day 7, the inner part of some spikelets was covered by *U. virens* mycelia, and the color of the base of the grains turned whitish (Figure 1C). On day 11, the fungal mass on a few infected spikelets protruded from the gap between the palea and lemma (Figures 1D,F). Eventually, false smut balls covered with yellow chlamydospores emerged at approximately 21 days (Figures 1E,G). Interestingly, under the same inoculation conditions, the average number of diseased grains of Guichao2 (indica rice) was higher than that of Zhejing99 (japonica rice), and the disease incidence rate of Guichao2 was also higher than that of Zhejing99 (japonica rice) (Table 1). However, this differed from natural conditions under which the susceptibility rate of Zhejing99 is higher than that of Guichao2.

**Table 1:** Incidence of rice false smut in indica and japonica rice.

Rice variety	Incidence rate/%		Average number of diseased grains
	Inoculation condition	Natural condition	
Guichao2 (indica)	73.33	33.5	16.2
Zhejing99 (japonica)	46.67	60.1	10.1

### 3.2 Transcriptome sequencing and DEG identification in *Ustilaginoidea virens* during infection of indica and japonica rice

To identify the pathogenic mechanism of *U. virens*-infected indica and japonica rice, transcriptome sequencing was performed using Illumina HiSeq 2500 system. A total of 753,074,444 clean reads and 112.97 Gb clean bases were generated from the 15 samples (Supplementary Table S1). The mapped reads of each sample with the *U. virens* reference genome were no less than 86.60%. The average sequencing base error rate of the corresponding QC data was less than 0.03%, and the sequencing data (Q20 > 98% and Q30 > 95%) showed excellent quality. These results indicate that the sequencing quality was high and suitable for further transcriptome analysis.

**Figure 2:**
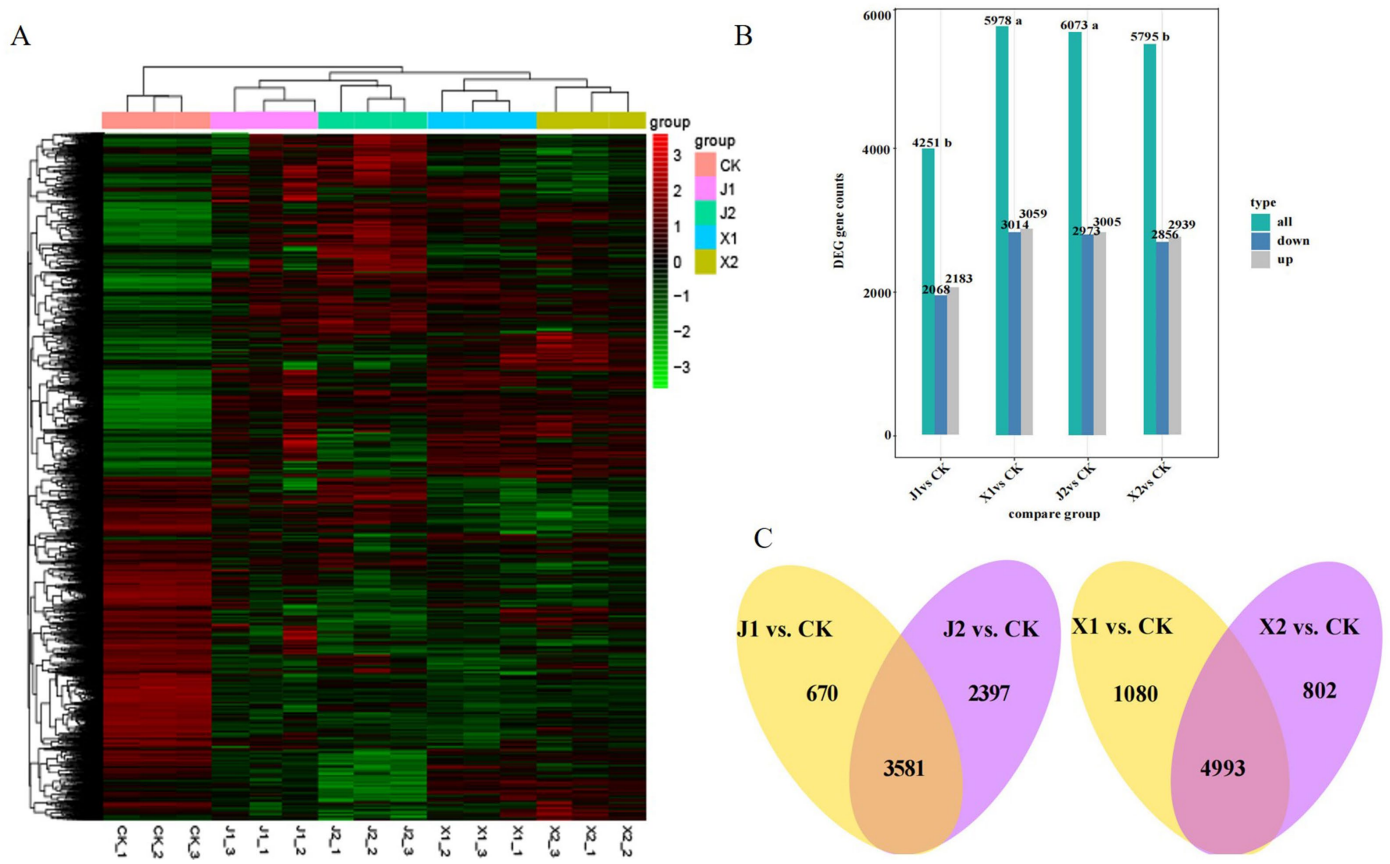
Analysis of DEG expression in *Ustilaginoidea virens* after infecting indica and japonica rice. **(A)** Heatmap of DEGs compared between different groups. **(B)** Number of DEGs identified in the four comparison groups. **(C)** Venn diagram of DEGs within the X group and within the J group.

The reliability and uniformity of the biological samples were verified by a correlation analysis. The correlation coefficient (R^2^) of the three biological replicates was greater than 0.92, indicating that the biological samples had high repeatability, a low systematic error, and high feasibility (Supplementary Figure S1A). Moreover, a heatmap cluster analysis showed that the expression of gene clusters was distinguishable among the five treatment groups (Figure 2A), which was consistent with the results of PCA (Supplementary Figure S1B). Subsequently, using a cutoff of |log2 fold-change| ≥ 1 and *p* < 0.05, DEGs were identified by comparing each of the sample groups to the control group (X1 vs. CK, X2 vs. CK, J1 vs. CK, and J2 vs. CK). As shown in Figure 2B, a total of 7,816 significant DEGs were identified. There were 6,073, 5,795, 4,251, and 5,978 DEGs in the X1 vs. CK, X2 vs. CK, J1 vs. CK and J2 vs. CK groups, respectively. At 5 d, the number of DEGs in X1 was significantly higher than in J1, whereas at 7 d, the number of DEGs in J2 was higher than in X2, indicating that the gene expression of *U. virens* differed when infecting indica rice and japonica rice at different time points. Venn diagram analysis was used to identify the DEGs that were present within the X and J group (Figure 2C). Among them, 4,993 DEGs were found only in the X group, while 3,581 DEGs were shared by the J1 group, indicating that the pathogenic mechanism of *U. virens* was more complex in indica rice than in japonica rice.

### 3.3 KEGG pathway analysis

**Figure 3:**
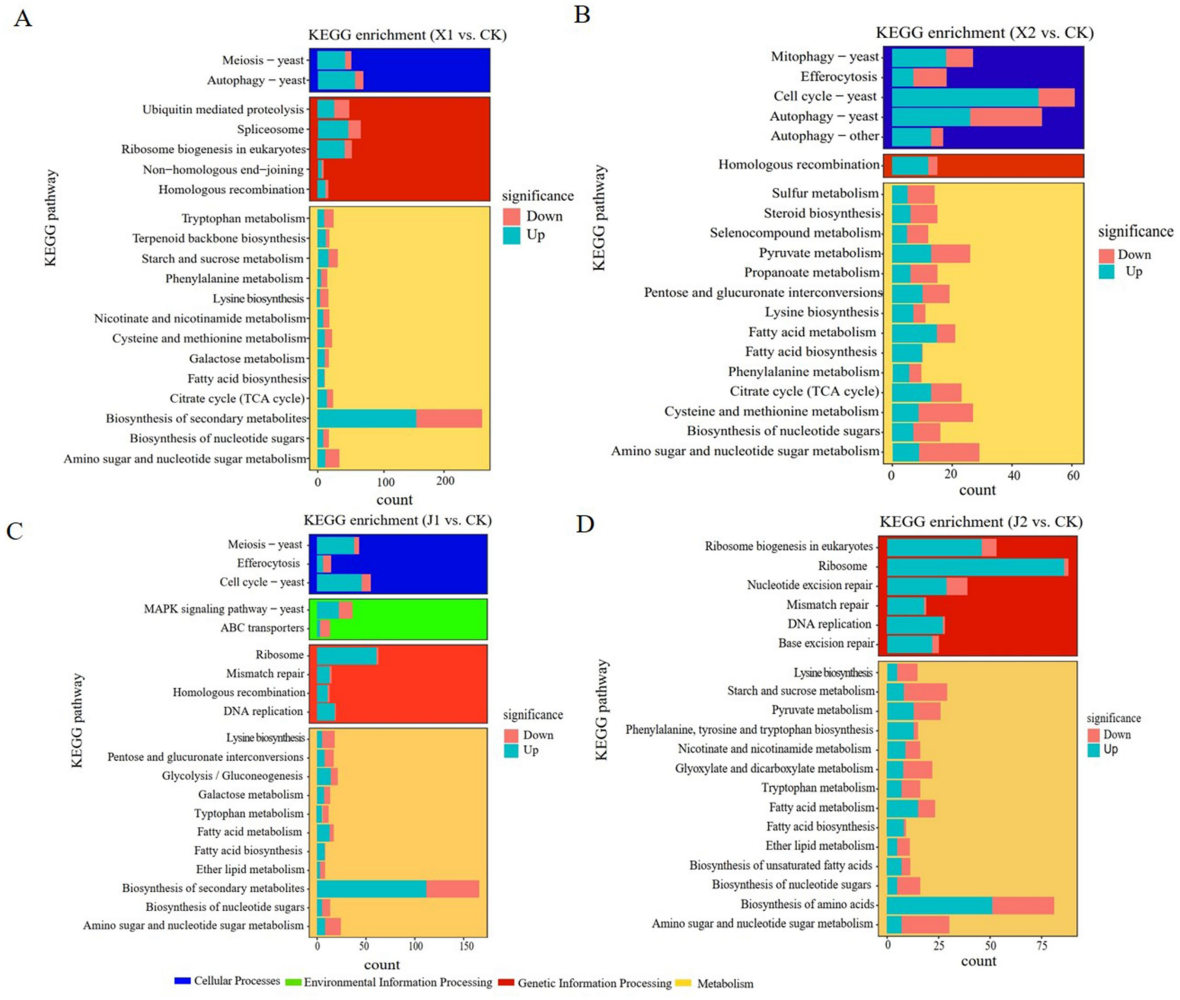
KEGG functional classification of differentially expressed genes. **(A)** X1 vs. CK; **(B)** X2 vs. CK; **(C)** J1 vs. CK; and **(D)** J2 vs. CK.

A pathway enrichment analysis using the KEGG database was performed on the DEGs of the four pairwise groups to further elucidate the pivotal metabolic pathways of *U. virens* infected indica and japonica rice. The results showed that the DEGs (1345) of *U. virens* in indica rice after 5 and 7 d of infection were concentrated in 100 metabolic pathways in three categories, namely cellular processes, genetic information processing and metabolism. In X1, the main significantly enriched metabolic pathways included phenylalanine metabolism, tryptophan metabolism, autophagy – yeast, starch and sucrose metabolism, cysteine and methionine metabolism, and lysine biosynthesis (Figure 3A). In X2, the main significantly enriched metabolic pathways included autophagy–yeast, tryptophan metabolism, lysine biosynthesis, phenylalanine metabolism, citrate cycle, and selenocompound metabolism (Figure 3B). The common significantly enriched metabolic pathways in both X1 and X2 were autophagy–yeast, tryptophan metabolism, cysteine and methionine metabolism, lysine biosynthesis, phenylalanine metabolism, and the citrate cycle. The DEGs (1333) of *U. virens* in japonica rice after 5 and 7 d of infection were involved in 100 metabolic pathways in four categories, namely cellular processes, environmental information processing, genetic information processing and metabolism. The metabolic pathways that were significantly enriched mainly included ribosomes, mitogen-activated protein kinase (MAPK) signaling pathway—yeast, ABC transporters, lysine biosynthesis, tryptophan metabolism, and fatty acid metabolism in J1 (Figure 3C). The metabolic pathways that were significantly enriched included ribosomes, lysine biosynthesis, starch and sucrose metabolism, fatty acid metabolism, and tryptophan metabolism in X2 (Figure 3D). The common significantly enriched metabolic pathways in both J1 and J2 were ribosomes, fatty acid metabolism, tryptophan metabolism, and lysine biosynthesis. The KEGG analysis results showed that the significantly enriched pathways of the differentially expressed genes of *U. virens* after infecting indica and japonica rice differed.

### 3.4 Analysis of differentially expressed genes in *Ustilaginoidea virens* after infecting indica and japonica rice

#### 3.4.1 Analysis of differentially expressed transcription factors

Transcription factors (TFs) are important proteins that regulate gene transcription initiation and therefore gene expression by binding to target gene promoter sequences, and thus influencing the growth, development, stress, and disease state of plant pathogens (Song et al., 2021). In this study, the type and number of differentially expressed TFs in *U. virens* after infecting indica and japonica rice were analyzed. The results showed the number of differentially expressed genes from the TF family was 82, 75, 56, and 76 in *U. virens* after infecting indica and japonica rice at 5 and 7 d (Table 2). TFs expression in *U. virens* was significantly affected on the 5th day after infecting indica rice, and the type and number of differentially expressed TFs reached their maximum at this time. We also analyzed the expression of certain differentially expressed TFs related to the pathogenicity of plant pathogens in each sample. The type and number of DEGs from TF families (i.e., C2H2, bHLH, MYB, and bZIP) related to pathogenicity in *U. virens* were significantly increased after infecting indica and japonica rice at 5 d. The number of upregulated genes in these families was 4, 4, 7, and 4 after infecting indica rice and 7, 2, 2, and 2 after infecting japonica rice.

**Table 2:** Changes in transcription factor expression in *Ustilaginoidea virens* after infecting indica and japonica rice.

TF	X1 vs. CK		X2 vs. CK		Total	J1 vs. CK		J2 vs. CK		Total
	Up	Down	Up	Down		Up	Down	Up	Down	
C2H2	4	9	8	9	19	7	5	6	11	20
bHLH	4	5	4	4	10	2	3	4	7	11
MYB	7	3	5	2	10	2	2	5	3	8
bZIP	4	5	4	4	9	2	4	2	5	8
GATA	3	2	3	3	7	2	1	1	3	5
Homeobox	4	2	4	1	6	4	2	3	2	7
ZBTB	1	2	1	1	3	1	3	2	2	5
HMG	1	1	1	1	3	0	1	1	1	2
ARID	3	0	3	0	3	2	0	1	0	2
CP2	0	1	0	1	1	0	1	0	1	1
CSD	1	0	0	0	1	1	0	0	0	1
Fork head	1	2	1	2	3	0	0	1	1	2
HSF	1	1	1	0	2	1	1	1	2	3
HTH	1	0	1	0	1	0	0	1	0	1
NDT80/PhoG	1	2	1	2	3	1	2	1	2	3
NF-YA	0	0	0	0	0	0	0	1	0	1
NF-YB	2	1	1	1	3	0	0	1	1	2
NF-YC	1	0	0	0	1	1	0	0	0	1
PC4	0	0	0	1	1	0	0	0	0	0
RFX	1	0	1	0	1	1	0	0	0	1
SRF	1	1	1	1	2	1	1	1	1	2
TEA	0	0	0	0	0	0	0	1	0	1
zf-LITAF-like	1	1	0	1	1	0	1	0	1	1
zf-MIZ	2	0	1	0	2	0	0	0	0	0
Total	44	38	41	34	92	28	27	33	43	88

#### 3.4.2 Analysis of MAPK signaling pathway DEGs

**Figure 4:**
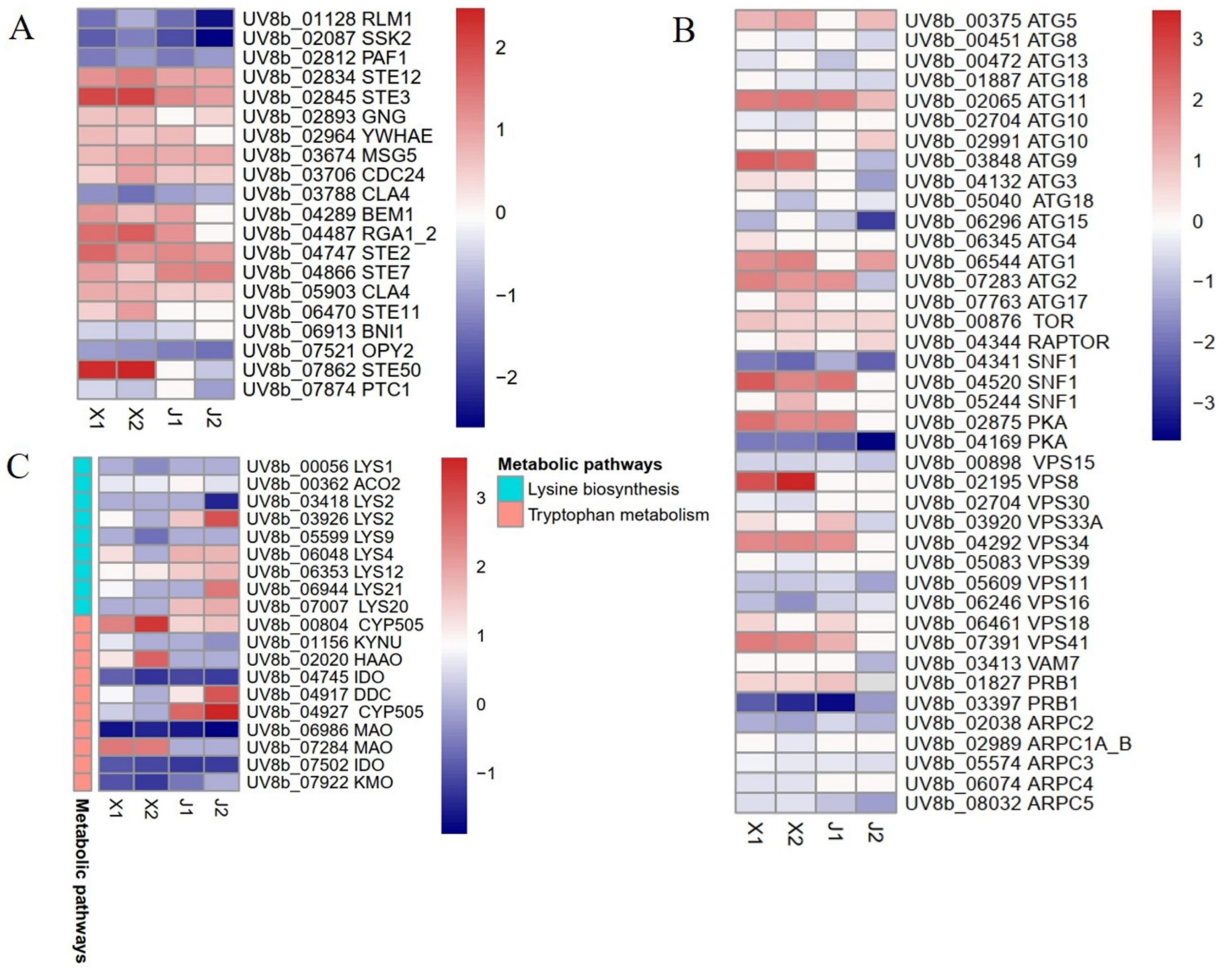
Differentially expressed gene analysis of *Ustilaginoidea virens* after infecting indica and japonica rice. **(A)** Analysis of MAPK signaling pathway DEGs. **(B)** Analysis of autophagy-related DEGs. **(C)** Analysis of amino acid metabolism DEGs.

The MAPK signaling pathway is an important regulator of plant pathogen infection in host plants. In this study, the DEGs in the MAPK pathway were analyzed, which showed that the expression levels of 20 genes in total had changed in *U. virens* after infecting indica and japonica rice. After infecting indica rice, 12 genes were upregulated, comprising six STE genes (STE2, STE3, STE7, STE11, STE12, and STE50), one tyrosine-protein phosphatase (MSG5) gene, one cell division control protein 24 (*CDC24*) gene, one guanine nucleotide-binding protein subunit gamma (GNG) gene, one Rho-type GTPase-activating protein (RGA) gene, one bud emergence protein 1 (BEM1) gene, and one 14–3-3 protein epsilon (YWHAE) gene. However, only eight genes were upregulated after infecting japonica rice, comprising four STE genes (STE2, STE3, STE7, and STE12), one MSG5 gene, one CDC24 gene, one GNG gene and one 14-3-3 protein epsilon (YWHAE) gene (Figure 4A; Supplementary Table S2). The results showed that the MAPK signaling pathway had an important effect on the pathogenicity of *U. virens*. In addition, the induction of STE genes indicated that there were differences in the regulatory mechanisms of the MAPK pathway in *U. virens* after infecting indica and japonica rice.

#### 3.4.3 Analysis of autophagy-related DEGs

Previous studies showed that autophagy has a regulatory effect on the growth development and pathogenicity of plant pathogens (Wang et al., 2019). Some autophagy regulatory factors, such as target of rapamycin (TOR) kinase, regulatory-associated protein of TOR (RAPTOP), protein kinase A (PKA), and SNF-1 kinase (SNF-1), regulate autophagy in cells under stress. In addition, some genes related to autophagy and other autophagy-related factors induce autophagy in cells. In this study, the autophagy regulatory factors and autophagy genes in the pathogen *U. virens* after infecting indica and japonica varieties were analyzed. The results showed that the expression levels of 40 genes related to autophagy changed after infection. Among the genes, the expression levels of seven autophagy regulatory factors changed. The expression levels of two negative regulators of autophagy (TOR and RAPTOP) were upregulated, while only two positive regulators (one PKA and one SNF1) were simultaneously upregulated at the two sampling time points after indica rice infection. The expression levels of 15 ATG genes changed, among which the expression levels of six genes (ATG1, ATG2, ATG3, ATG5, ATG9, and ATG11) were upregulated at the two time points after indica rice infection. The expression of ATG11 was upregulated at the two time points after japonica rice infection. In addition, the expression levels of 10 genes related to the phosphatidylinositol 3-kinase complex (PtdIns3K) changed, with only three of the genes (VPS8, VPS33A, and VPS41) upregulated at the two time points after indica rice infection (Figure 4B; Supplementary Table S3).

#### 3.4.4 Analysis of amino acid metabolism DEGs

Studies have shown that amino acid synthesis and metabolism are necessary for vegetative growth, development, the infection cycle, and the pathogenicity of plant pathogens (Chen et al., 2014). Here, the DEGs in four amino acid metabolic pathways were analyzed: tryptophan metabolism, cysteine and methionine metabolism, lysine biosynthesis, and phenylalanine metabolism (Figure 4C; Supplementary Table S4). In tryptophan metabolism, the important regulatory genes that were induced after pathogen infection in indica rice were *CYP505*, *HAAO*, and *MAO*, and the genes that were upregulated after infecting japonica rice were *CYP505* and *DDC*. In the lysine synthesis pathway, the regulatory genes *LYS2*, *LYS4*, and *LYS20* were upregulated after infection in japonica rice but were inhibited or reduced after infection in indica rice. Therefore, the results of this study indicated that amino acid metabolic pathways had an important role in the pathogen infection of rice, but the pathogen infection mechanisms differed between indica and japonica rice.

### 3.5 RT-qPCR validation of DEGs

To verify the dependability of the RNA-seq data, we randomly chose 10 candidate genes to confirm the sequencing results. Similar to the results of the RNA-seq method, the 10 DEGs analyzed by qPCR-PCR differed significantly after infecting indica and japonica rice (Supplementary Table S5). Metabolome quality control of *Ustilaginoidea virens* after infection of indica and japonica rice.

Metabolites are considered the downstream products of both the genome and its interactions with the environment (Wang et al., 2022). To explore the differences in the metabolite levels of *U. virens* after infecting rice, the changes in the metabolites of the mycelium of *U. virens* infections of indica (X) and japonica (J) rice were identified using a UHPLC-QE-MS detection platform with positive (POS) and negative (NEG) ion modes. QC is a necessary step for obtaining stable and accurate metabolome data. As shown in the Supplementary Figures S2A,B, the Pearson correlation coefficient between QC samples was close to 1 under both POS and NEG modes, indicating that the entire detection process was stable and that the data quality was high (Supplementary Figures S2A,B). Consistently, PCA revealed that the four groups were well separated under both POS and NEG modes. The two principal components explained 47.46 and 55.90 of the variability using POS and NEG modes, respectively, with differences mainly distinguished by PC1 (Supplementary Figures S2C,D).

### 3.6 Differential metabolite analysis

**Figure 5:**
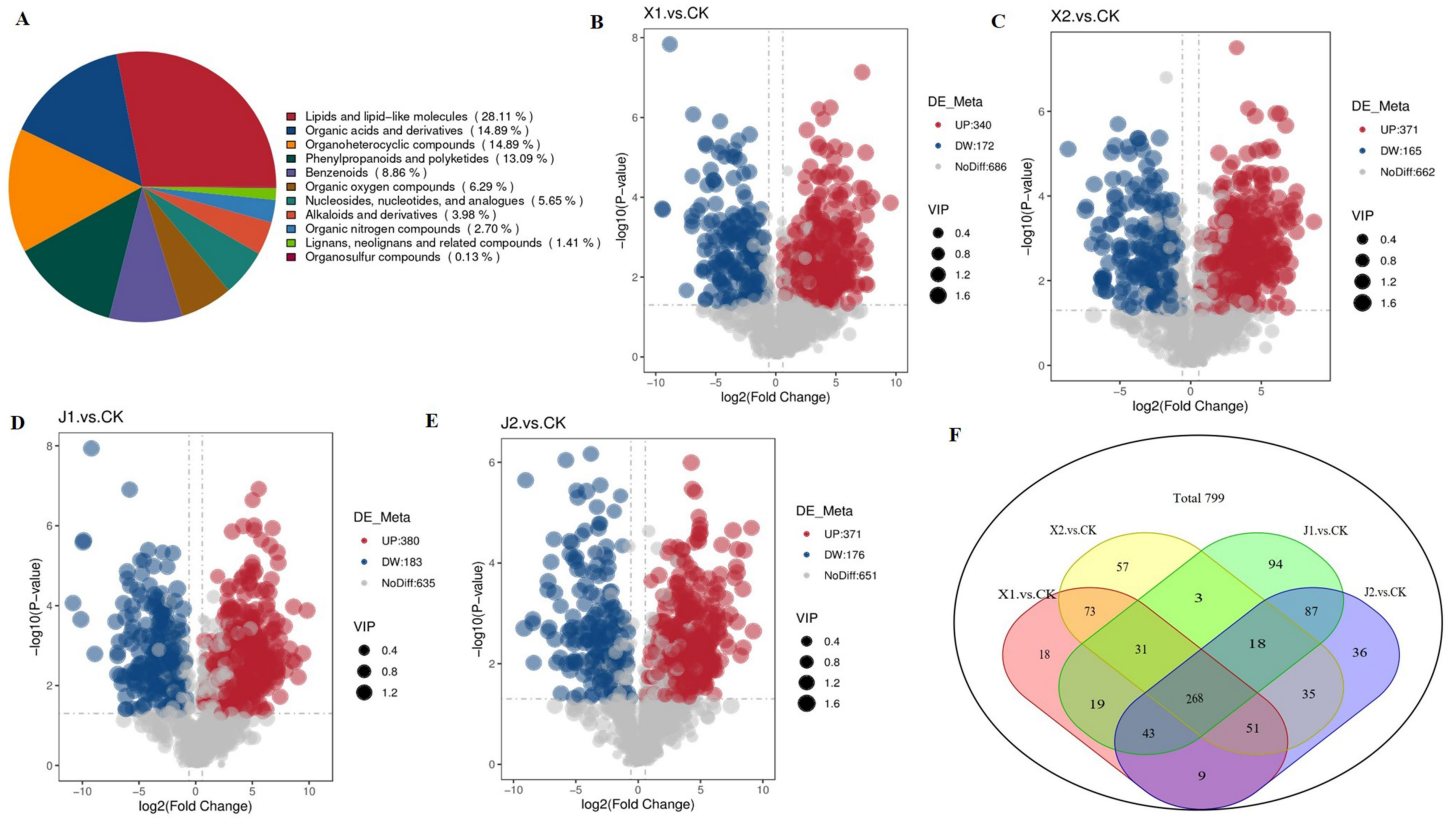
Analysis of DAM expression in *Ustilaginoidea virens* after infecting indica and japonica rice. **(A)** Pie chart of DAMs classification. **(B–E)** Volcano plots of DAMs in each of the four comparison groups. **(F)** Venn diagram of DAMs in all comparison groups.

Using VIP ≥ 1 and *p* < 0.05 as threshold, a total of 799 DAMs were detected across all samples. The DAMs were mainly classified into 11 categories, including 219 lipids and lipid-like molecules, 116 organic acids and derivatives, 116 organoheterocyclic compounds, 102 phenylpropanoids and polyketides, 69 benzenoids, and 49 organic oxygen compounds (Table 3; Figure 5A). Among them, there were 512 DAMs in the X1 vs. CK comparison (340 upregulated and 172 downregulated) (Figure 5B), 536 metabolites in the X2 vs. CK comparison (371 upregulated and 165 downregulated) (Figure 5C), 563 metabolites in the J1 vs. CK comparison (380 upregulated and 183 downregulated) (Figure 5D), and 547 metabolites in the J2 vs. CK comparison (371 upregulated and 176 downregulated) (Figure 5E). A Venn diagram analysis showed that 268 DAMs were common to the four comparison groups (Figure 5E). Additionally, 361 and 372 DAMs were shared by the X1 and J1 groups and by the X2 and J2 groups, respectively (Figure 5F).

**Table 3:** Types and quantities of differential accumulated metabolites.

Species of metabolites	Number of metabolites
Lipids and lipid-like molecules	219
Organic acids and derivatives	116
Organoheterocyclic compounds	116
Phenylpropanoids and polyketides	102
Benzenoids	69
Organic oxygen compounds	49
Nucleosides, nucleotides, and analogs	44
Alkaloids and derivatives	31
Organic nitrogen compounds	21
Lignans, neolignans and related compounds	11
Organosulfur compounds	1
All	799

**Figure 6:**
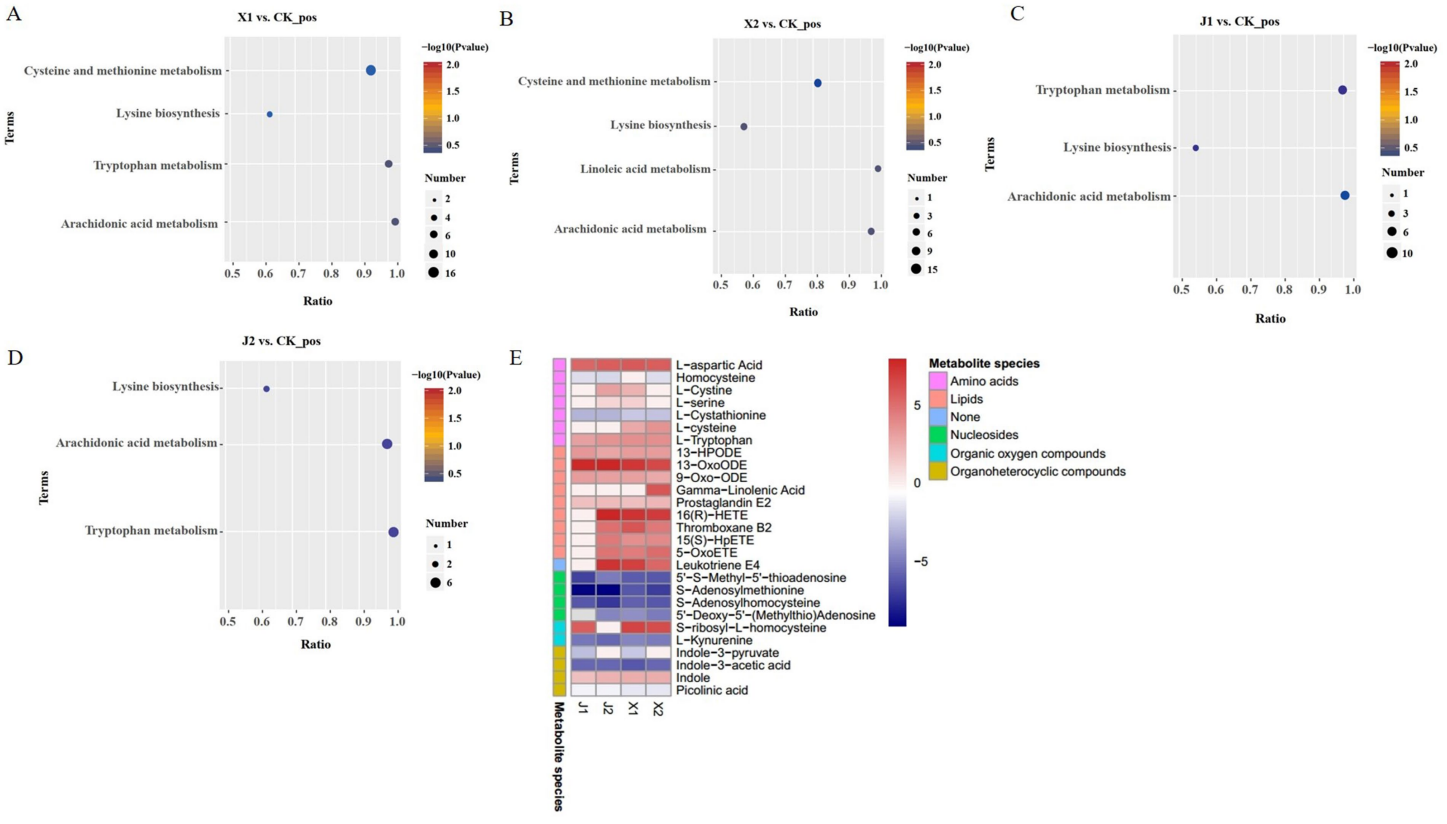
Differential metabolite analysis. **(A–D)**. KEGG pathway analysis of the DAMs in **(A)** X1 vs. CK, **(B)** X2 vs. CK, **(C)** J1 vs. CK, and **(D)** J2 vs. CK. **(E)** Heatmaps of DAMs.

KEGG enrichment analysis was performed to identify the significantly enriched KEGG pathways of the DAMs. The results showed that the DAMs were mainly significantly enriched in five pathways: cysteine and methionine metabolism, tryptophan metabolism, arachidonic acid metabolism, lysine biosynthesis, and linoleic acid metabolism. Among them, arachidonic acid metabolism and lysine biosynthesis were enriched in the four infected groups compared with the CK group (Figures 6A–D). Cysteine and methionine metabolism were significantly enriched in the X1 vs. CK and X2 vs. CK groups (Figures 6A,B). Tryptophan metabolism was significantly enriched in the J1 vs. CK and J2 vs. CK groups (Figures 6C,D). Therefore, the results suggested that the differences in *U. virens* after infecting indica rice and japonica rice could be caused by cysteine, methionine, and tryptophan metabolism.

A total of 28 types of DAMs, comprising nine lipids, four nucleosides, nine amino acids, two organic oxygen compounds, and four organoheterocyclic compounds, were enriched in five KEGG pathways. Among these DAMs, 14 had higher levels and 10 had lower levels in the X1 vs. CK and X2 vs. CK groups than in the other comparison groups (Figure 6E). There were 7 DAMs with higher levels and 10 DAMS with lower levels in the J1 vs. CK and J2 vs. CK groups than in the other comparison groups. Therefore, these amino acids, lipids, and nucleic acids have important roles in the infection process of *U. virens*. In addition, these results indicate that metabolite levels in *U. virens*-infected indica and japonica rice differed.

### 3.7 Transcriptome and metabolome modulation of *Ustilaginoidea virens* after infecting indica and japonica rice

**Figure 7:**
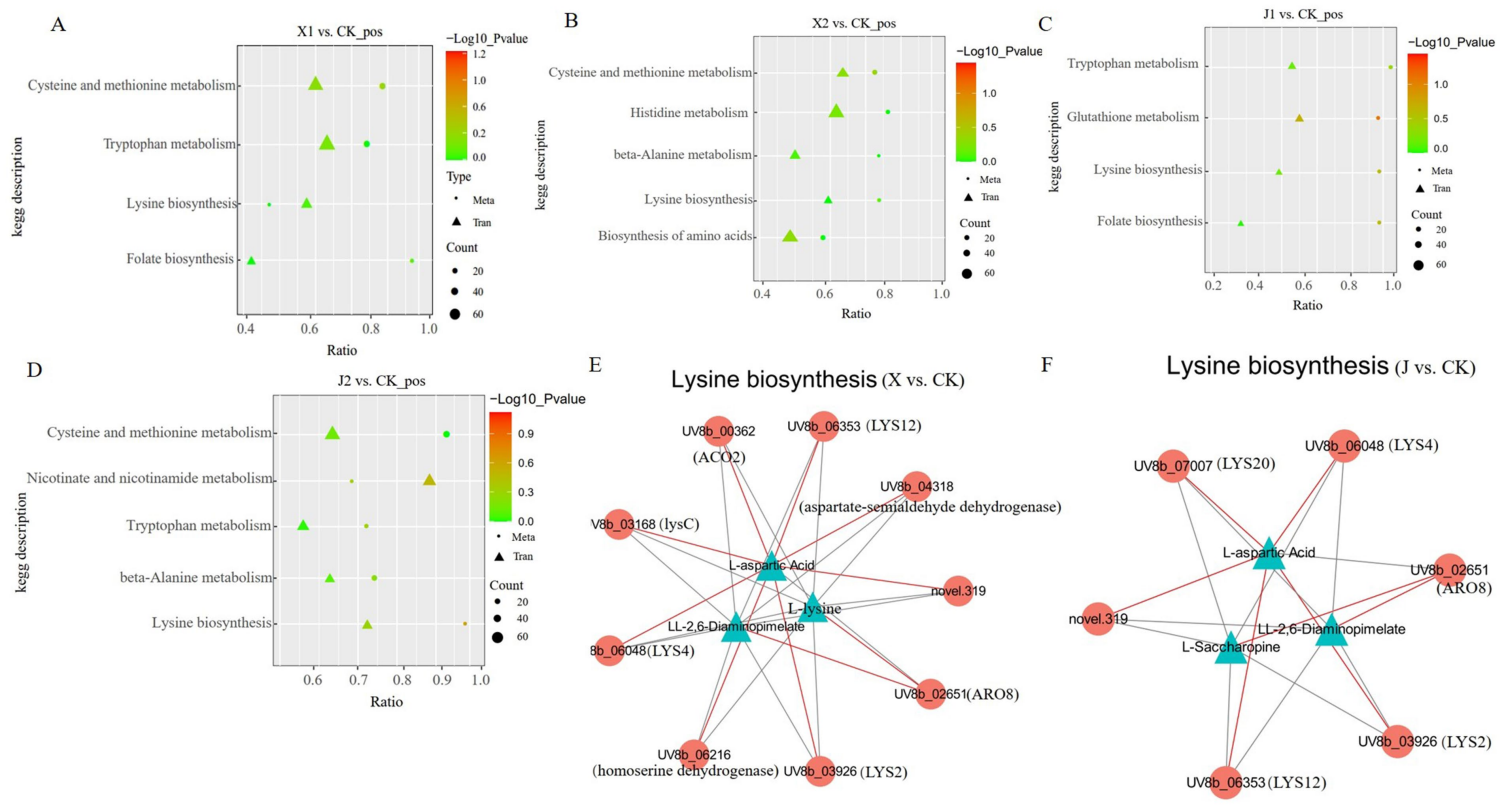
Correlation analysis among the DEGs and DAMs of *Ustilaginoidea virens* after infecting indica and japonica rice. **(A–D)** Graphs of the DEGs and DAMs that were significantly enriched in the same KEGG pathway among the four groups (X1, X2, J1, and J1) in comparison to the control. **(E,F)** Co-expression network analysis between the DEGs and DAMs.

A combined transcriptome and metabolome analysis was then conducted to further elucidate the function of the DEGs and the DAMs of *U. virens* after infecting indica and japonica rice. Many DEGs and DAMs were co-enriched in KEGG pathways, including cysteine and methionine metabolism, tryptophan metabolism, lysine biosynthesis, beta-alanine metabolism, glutathione metabolism, and phenylalanine metabolism (Figures 7A–D). Among them, cysteine and methionine metabolism were only enriched in *U. virens* after infecting indica rice (X1 vs. CK and X2 vs. CK), while tryptophan metabolism was only enriched in *U. virens* after infecting japonica rice (J1 vs. CK and J2 vs. CK). In addition, only lysine biosynthesis was significantly enriched in *U. virens* after infecting indica and japonica rice. According to these findings, the metabolite changes may be regulated by their respective genes either directly or indirectly which may be closely related to the mechanism by which *U. virens* infected indica and japonica rice.

Based on the Pearson correlation coefficients, a correlation network graph of the DEGs and DAMs was constructed to further investigate the gene regulatory network of *U. virens* after infecting indica and japonica rice. In this experiment, the DAMs and DEGs with a correlation greater than 0.8 were selected for a correlation network map of lysine biosynthesis. The results of the correlation study showed that three metabolites displayed a strong correlation with eight genes in the X vs. CK comparison. The metabolites included L-aspartic acid, L-lysine, and LL-2,6-diaminopimelate. The following eight related enzymes also correlated: ARO8 [EC: 2.6.1.57, 2.6.1.39, 2.6.1.27, 2.6.1.5], LYS12 [EC: 1.1.1.87], aspartate-semialdehyde dehydrogenase [EC: 1.2.1.11], lysC [EC: 2.7.2.4], LYS2 [EC: 1.2.1.95], LYS4 [EC: 4.2.1.36], homoaconitase [EC: 4.2.1], and homoserine dehydrogenase [EC: 1.1.1.3]. Among them, LYS2, LYS4, LYS12, lysC, ACO2, and aspartate-semialdehyde dehydrogenase were highly positively connected to L-aspartic acid (Figure 7E; Supplementary Table S6). Furthermore, the results showed that three metabolites, L-aspartic acid, L-saccharopine, and LL-2,6-diaminopimelate, strongly correlated with five genes in the J vs. CK comparison. These genes encoded five enzymes, LYS12 [EC: 1.1.1.87], LYS4 [EC: 4.2.1.36], ARO8 [EC: 2.6.1.57, 2.6.1.39, 2.6.1.27, 2.6.1.5], LYS20 [EC: 2.3.3.14], and LYS2 [EC: 1.2.1.95]. Among them, LYS2, LYS4, LYS12, and LYS20 were highly positively connected to L-aspartic acid (Figure 7F; Supplementary Table S7). The results indicated that the DEGs of the regulatory metabolites in the same metabolic pathway differed, which further confirmed that the pathogenic mechanisms by which *U. virens* infected indica and japonica rice differed.

## 4 Discussion

RFS has become one of the major fungal diseases in all rice-growing areas, seriously threatening the food security of rice-consuming nations. It has been reported that different rice varieties have different susceptibility levels to RFS. Generally, the susceptibility trend is as follows: glutinous varieties > japonica varieties > indica varieties; late rice > early rice; and hybrid rice > conventional rice (Chen et al., 2019; Fu et al., 2022). However, in this study, under the same inoculation conditions, the incidence rate of Guichao2 (indica rice) was higher than that of Zhejing99 (japonica rice), which was different from the disease trend reported under natural conditions. The reason for this phenomenon may be related to the difference between syringe inoculation and natural field infection, or to the fact that indica and japonica have distinct genetic backgrounds. Given this, the aim of this study was to explore the pathogenic mechanism of *U. virens* infection of indica and japonica rice based on two omics, transcriptomics and metabolomics.

Integrated transcriptome and metabolome analyses have been applied to explore a variety of important problems, such as the molecular mechanism of plant responses to pathogen infection (Sana et al., 2010) and the regulatory mechanisms of pathogenic fungi growth and development (Xu et al., 2018). In this study, transcriptome and metabolome analyses were used to study the changes in gene expression and metabolite levels in *U. virens* after infecting indica and japonica rice to explore the molecular pathogenic mechanism in different rice varieties. First, comparative transcriptome analyses showed that the expression levels of 7,816 genes were significantly changed in *U. virens* after infecting indica and japonica rice. Based on the KEGG analysis, the significantly enriched metabolic pathways differed between *U. virens*-infected indica rice and japonica rice. The DEGs in *U. virens* after infecting indica rice were significantly enriched in starch and sucrose metabolism, cell cycle—yeast, mitophagy—yeast, tryptophan metabolism, cysteine and methionine metabolism, lysine biosynthesis, phenylalanine metabolism, and the citrate cycle. However, the DEGs in *U. virens* after infecting japonica rice were significantly enriched in the MAPK signaling pathway—yeast, lysine biosynthesis, tryptophan metabolism, biosynthesis of secondary metabolites, and amino sugar and nucleotide sugar metabolism. Previous studies reported that metabolic pathways such as mitophagy—yeast, tryptophan metabolism, lysine biosynthesis, and MAPK signaling pathway were closely related to the vegetative growth, development, and pathogenicity of plant pathogens.

TFs are crucial in plant pathogen cell growth, infection, and invasion of the host (Cao et al., 2019). In this study, the expression levels of 24 families of TFs changed after *U. virens* infection of indica and japonica rice varieties. Among them, the expression levels of C2H2, bHLH, MYB, *bZIP*, and homeobox genes varied significantly at certain time points after *U. virens* infection of indica rice and japonica rice. Previous studies reported that TFs such as C2H2, bHLH, bZIP, and homeobox affected the vegetative growth, spore production, infection, and pathogenicity of *U. virens* (Chen et al., 2021; Qu et al., 2022; Yu et al., 2019). Therefore, the results indicated that these TFs may be part of the molecular mechanisms responsible for the different infection patterns of *U. virens* in indica rice and japonica rice.

MAPK signaling pathways are important in the growth, development, propagation, and pathogenicity of various pathogenic plant fungi (Zhao and Xu, 2005). The proteins encoded by the STE gene family, such as STE20, STE12, STE11, STE7, and STE50, are crucial in multiple signaling processes. Consistent with previous findings, MAPK was the main signal pathway in *U. virens* after infection in this study. We found that the levels of six STE genes significantly changed in *U. virens* after infection of indica and japonica rice. Among them, STE11 and STE50 were significantly upregulated only after indica rice infection. Previous studies showed that STE50 directly interacted with STE11 to transmit upstream signals to STE11, thereby activating the downstream MAPK cascade reaction (Sharmeen et al., 2019). Therefore, the results indicated that the MAPK signaling pathway and STE genes promote the infection and pathogenicity process of *U. virens*. Moreover, differences in *U. virens* infection between indica and japonica rice may be mainly caused by STE11 and STE50.

In recent decades, studies have shown that autophagy affects the virulence of pathogenic plant fungi (Nguyen et al., 2011). The autophagy process is complex and regulated by autophagy regulatory factors, both negative regulatory factors (TOR kinase and RAPTOP) and positive regulatory factors (PKA and SNF-1), and autophagy-related genes, such as ATG and those of the PtdIns3K complex (Nguyen et al., 2011; Puente et al., 2016). In this study, two positive regulators of autophagy (PKA and SNF1) and two negative regulators (TOR and RAPTOP) were upregulated in *U. virens* after infection of indica rice. These results are inconsistent with previous research (Fu et al., 2023). Therefore, we speculated that *U. virens* activated the autophagy pathway by activating the expression of the positive regulatory factors PKA and SNF1. Here, six ATG genes (ATG1, ATG2, ATG3, ATG5, ATG9, and ATG11) were upregulated after indica rice infection, but only ATG11 was upregulated after japonica rice infection. Previous studies reported that the autophagy-related gene *UvAtg8* was associated with the spore production, stress response, and pathogenicity of *U. virens* (Meng et al., 2020). Presently, several ATG genes have been determined to be necessary for the pathogenicity of fungal plant pathogens (Nadal and Gold, 2010; Yanagisawa et al., 2013). Taken together, our results suggest that the expression of genes related to autophagy regulation may affect the pathogenicity of *U. virens*. Furthermore, the autophagy mechanism of *U. virens* in infected indica and japonica rice differed.

Many studies have shown that amino acid metabolism plays an important role in mycelium vegetative growth, the infection cycle, and the pathogenicity of pathogens (Aron et al., 2021). It was reported that the synthesis and metabolism of many amino acids, including “lysine biosynthesis, cysteine and methionine metabolism, tryptophan metabolism and phenylalanine metabolism,” are crucial for the pathogenicity of plant pathogens (Chen et al., 2014; Zhang et al., 2015). In this study, DEG analysis revealed that the expression levels of many genes involved in these four metabolic pathways significantly changed in *U. virens* after indica and japonica rice infection. A metabolome analysis found that the levels of the metabolites L-tryptophan, L-aspartic acid, L-cysteine, L-saccharopine, and L-serine, involved in four metabolic pathways, were also significantly changed in *U. virens* after infecting indica and japonica rice. However, the changes in these metabolite levels in response to *U. virens* infection of indica and japonica rice differed. For example, the level of L-cystine was upregulated after the fungus infected indica rice but was inhibited after infecting japonica rice. Previous studies showed that these metabolites were closely related to the pathogenicity of plant pathogens, such as *U. virens* (Fu et al., 2023), *Magnaporthe oryzae* (Chen et al., 2014), and *Fusarium graminearum* (Seong et al., 2005). Taken together, these results indicated that the genes and metabolites involved in amino acid metabolism were closely related to the virulence of *U. virens*. However, *U. virens* showed differences in the gene expression and metabolites of amino acid metabolism after infecting indica and japonica rice.

Many studies have successfully shown that the combined analysis of transcriptomic and metabolomic data can help reveal the complexity of biological systems and their interaction networks (Liu et al., 2024; Xu et al., 2018). In this study, to obtain an in-depth analysis of the changes in the pathogen *U. virens* after infecting indica and japonica rice, a combined analysis of the transcriptome and metabolome was performed. Three amino acid metabolic pathways (cysteine and methionine metabolism, tryptophan metabolism, and lysine biosynthesis) were found to be significantly co-enriched in the combined analysis. Among them, the metabolism of cysteine and methionine was enriched in *U. virens* after infecting indica rice, while tryptophan metabolism was enriched in *U. virens* after infecting japonica rice. In addition, lysine biosynthesis was significantly enriched in *U. virens* after infecting indica and japonica rice. Therefore, we speculated that these three amino acid metabolic pathways may be the key pathways underlying the pathogenicity of *U. virens*, which is consistent with previous studies (Fu et al., 2023). Furthermore, the network correlation analysis indicated that the DEGs of the regulatory metabolites in the same metabolic pathways were different, which further confirmed that the pathogenic mechanism by which *U. virens* infected indica and japonica rice differed. Therefore, different DAMs regulated by different DEGs may be the cause of the pathogenicity difference in *U. virens* after infecting indica and japonica rice.

## 5 Conclusion

This study revealed the pathogenic mechanisms of *U. virens* in indica and japonica rice through transcriptome and metabolomic analyses. Comparative transcriptome analysis showed that the DEGs involved in the MAPK signaling, autophagy, and amino acid metabolic pathways exhibited different expression patterns in *U. virens* after infecting indica and japonica rice varieties. Metabolome analysis showed that the significantly enriched pathways of DAMs mainly included amino acids, lipids, and nucleic acids in *U. virens* after infection. Moreover, the accumulation patterns of these metabolites showed significant differences in *U. virens* after infecting indica and japonica rice. These results indicated that the pathogenic mechanisms of *U. virens*, which differed between indica and japonica rice, might be caused by these metabolic pathways, genes, and metabolites. Moreover, integrated transcriptome and metabolome analysis further indicated that different DAMs regulated by different DEGs may be the cause of the difference in the pathogenicity of *U. virens* after infecting indica and japonica rice. These findings have enhanced our understanding of the molecular mechanisms by which *U. virens* causes disease in different rice varieties.

## Data Availability

The datasets presented in this study can be found in online repositories. The names of the repository/repositories and accession number(s) can be found in the article/Supplementary material.

## Supplementary material

Supplementary Figure S1 Quality analysis of transcriptome data. **(A)** Heatmap of the Pearson correlation between the samples. **(B)** Principal component analysis (PCA) plots of the samples.

Supplementary Figure S2 Quality control analysis of the metabolomic data. **(A)** Correlation analysis of the QC samples. **(B)** PCA of the total sample.
