# Supplementary figures and images for: Transcriptomic and metabolomic analyses unravel the different pathogenic mechanisms of Ustilaginoidea virens in indica and japonica rice
Source: Front Microbiol. 2025 Oct 17;16:1680221. doi: 10.3389/fmicb.2025.1680221 (PMC12576890; doi:10.3389/fmicb.2025.1680221)

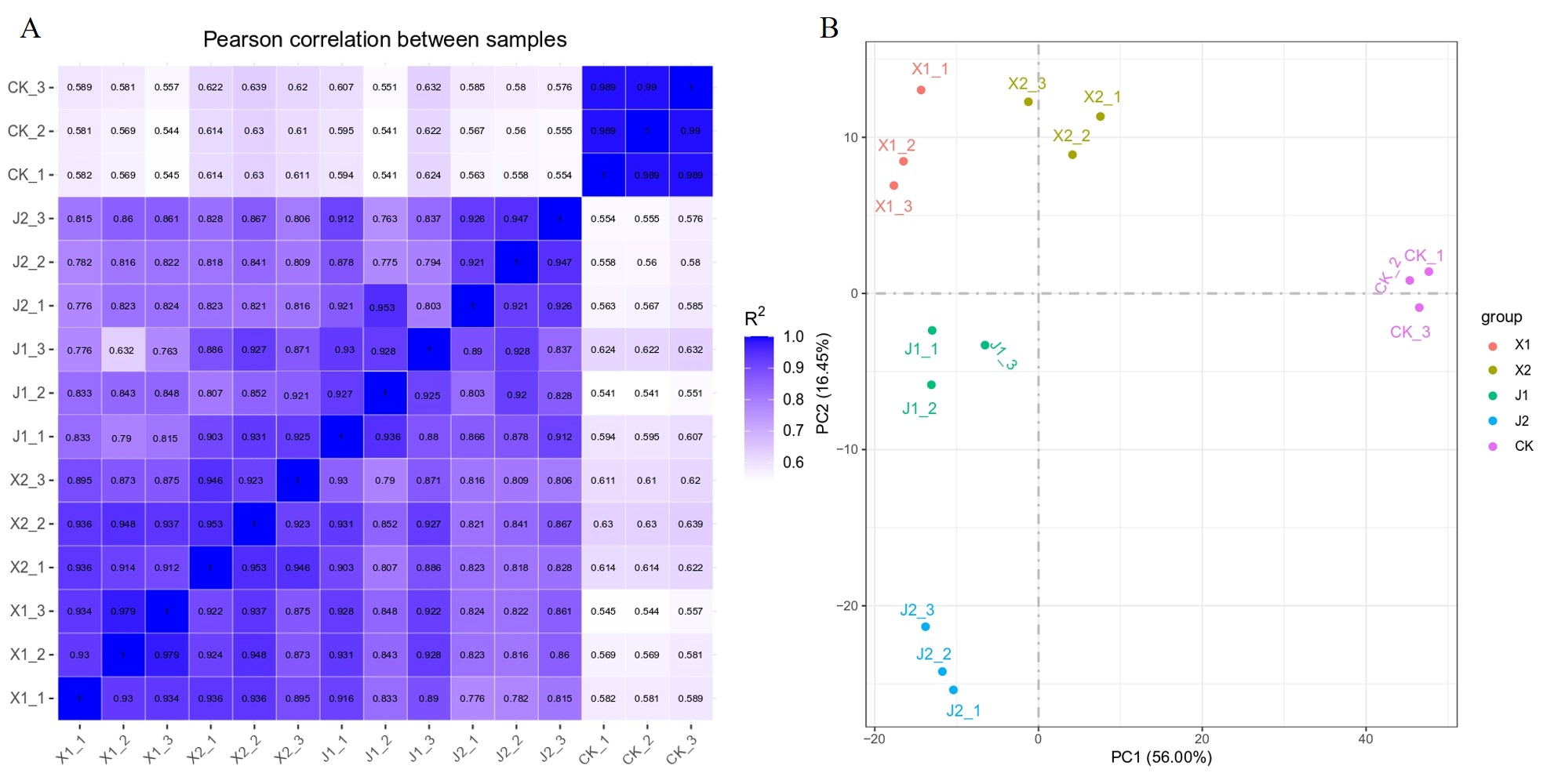

Supplement: Supplementary Figure S1 — Quality analysis of transcriptome data. (A) Heatmap of the Pearson correlation between the samples. (B) Principal component analysis (PCA) plots of the samples. [file Image_1.jpeg]

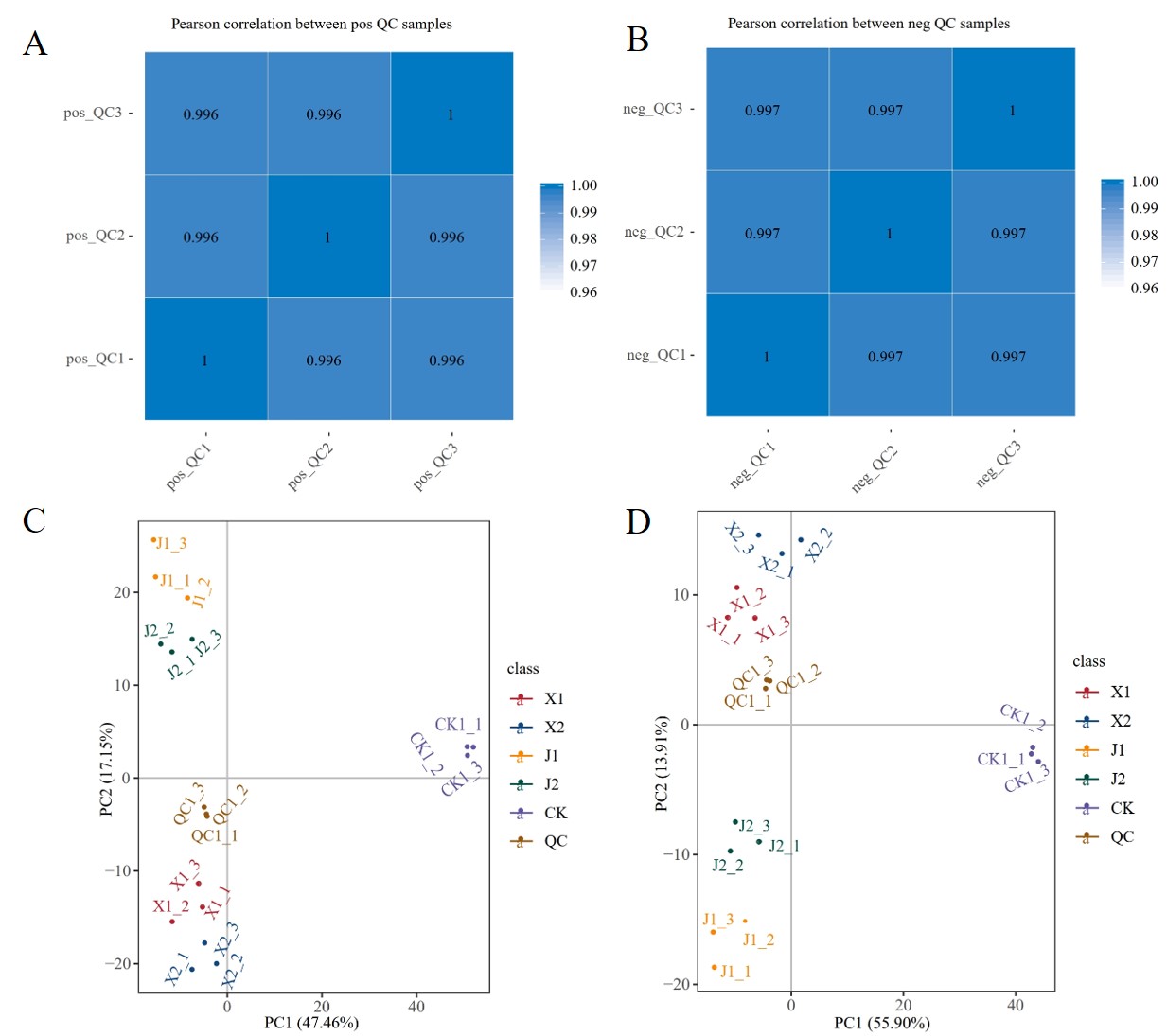

Supplement: Supplementary Figure S2 — Quality control analysis of the metabolomic data. (A) Correlation analysis of the QC samples. (B) PCA of the total sample. [file Image_2.jpeg]
